# CONCORDE: A phase I platform study of novel agents in combination with conventional radiotherapy in non-small-cell lung cancer

**DOI:** 10.1016/j.ctro.2020.09.006

**Published:** 2020-09-22

**Authors:** Gerard M. Walls, Jamie B. Oughton, Anthony J. Chalmers, Sarah Brown, Fiona Collinson, Martin D. Forster, Kevin N. Franks, Alexandra Gilbert, Gerard G. Hanna, Nicola Hannaway, Stephen Harrow, Tom Haswell, Crispin T. Hiley, Samantha Hinsley, Matthew Krebs, Geraldine Murden, Rachel Phillip, Anderson J. Ryan, Ahmed Salem, David Sebag-Montefoire, Paul Shaw, Chris J. Twelves, Katrina Walker, Robin J. Young, Corinne Faivre-Finn, Alastair Greystoke

**Affiliations:** a Patrick G Johnston Centre for Cancer Research, Queen’s University Belfast, Northern Ireland, UK; b Leeds Institute of Clinical Trials Research, University of Leeds, England, UK; c Institute of Cancer Sciences, University of Glasgow, Scotland, UK; d Department of Oncology, UCL Cancer Institute, England, UK; e St James’ Institute of Oncology, University of Leeds, England, UK; f Sir Peter MacCallum Department of Oncology, University of Melbourne, Australia; g Newcastle University, Newcastle upon Tyne, England, UK; h The Beatson West of Scotland Cancer Centre, Glasgow, Scotland, UK; i Patient and Public Involvement Advocacy, UK; j Faculty of Biology, Medicine and Health, University of Manchester, England, UK; k Oxford Institute for Radiation Oncology, University of Oxford, Oxford, England, UK; l The Christie NHS Foundation Trust/University of Manchester, Manchester, England, UK; m Velindre University NHS Trust, Cardiff, Wales, UK; n Academic Unit of Clinical Oncology, Weston Park Hospital, Sheffield, England, UK

**Keywords:** ATM, Ataxia telangiectasia mutated, ATR, Ataxia telangiectasia and Rad3 related, cfDNA, Cell-free DNA, CRT, Chemoradiotherapy, CT, Computed tomography, CTCAE, Common terminology criteria for adverse events, CTRad, Clinical and Translational Radiotherapy Research Working Group, DDRi, DNA damage response inhibitor, DLT, Dose limiting toxicity, DNA, Deoxyribonucleic acid, DNA-PK, DNA-dependent protein kinase, ECOG, Eastern Cooperative Oncology Group, EORTC, European Organisation for Research and Treatment of Cancer, ICRU, International Commission on Radiation Units and Measurements, IMPs, Investigational medicinal products, LA, Locally advanced, MRC, Medical Research Council, NCRI, National Cancer Research Institute, NSCLC, Non-small cell lung cancer, PARP, Poly (ADP-ribose) polymerase, PET, Positron emission tomography, PFS, Progression free survival, PROMs, Patient-reported outcome measures, RECIST, Response evaluation criteria in solid tumours, RP2D, Recommended phase II dose, RT, Radiotherapy, SACT, Systemic anti-cancer therapy, SRC, Safety review committee, TiTE-CRM, Time to event continual reassessment method, TNM, Tumour node metastasis, Non-small cell lung cancer, Sequential chemoradiotherapy, DNA damage repair inhibitor, Platform trial, Continual reassessment method

## Abstract

Lung cancer is the leading cause of cancer mortality worldwide and most patients are unsuitable for ‘gold standard’ treatment, which is concurrent chemoradiotherapy. CONCORDE is a platform study seeking to establish the toxicity profiles of multiple novel radiosensitisers targeting DNA repair proteins in patients treated with sequential chemoradiotherapy. Time-to-event continual reassessment will facilitate efficient dose-finding.

## Highlights

• Lung cancer is the leading cause of cancer mortality worldwide.
• Gold standard chemoradiotherapy is not deliverable for the majority of patients.
• Radiation-induced DNA damage repair is an actionable mechanism of radioresistance.
• Careful trial design is essential to elicit maximum impact on therapeutic index.
• CONCORDE is a platform study of novel drug/radiotherapy combinations in lung cancer.

## 1 Introduction

Lung cancer is the leading cause of cancer mortality worldwide, with over 46,000 new cases diagnosed annually in the UK alone [1] and the incidence is projected to increase, excluding any pending screen-detected caseload [2], [3]. The majority of cases are non-small cell histology (NSCLC), and approximately one quarter of patients present with stage III tumours [4].

Concurrent chemoradiotherapy (CRT) is recommended for fit patients with unresectable stage III disease [5]. Despite advancements in radiotherapy (RT) technology, 5-year survival remains low at approximately 32% [6]. Cytotoxic systemic anti-cancer therapy (SACT) concurrently with RT is not deliverable for the majority of patients due to tumour bulk or poor fitness [7] and is delivered sequentially rather than concurrently for almost two-thirds of UK cases [8]. Sequential treatment is associated with 5-year survival rates of only 10%, largely owing to higher rates of loco-regional failure [5].

Since RT dose escalation has failed to improve outcomes in locally advanced disease (LA-NSCLC), investigations of intensified schedules [9], [10], improved conformality [11], [12] and consolidative immunotherapy [13] have been undertaken recently. While the biology underpinning the intrinsic radioresistance of NSCLC remains incompletely defined, repair of radiation-induced DNA damage is considered a fundamental component [14]. These pathways are actionable [15], [16], [17], [18]: potent novel agents targeting DNA damage response pathways are becoming clinically available [19] and strategies for synergistic combination of systemic agents with RT are being realised across tumours [20], [21], [22], [23], [24]. Careful, efficient and multidisciplinary clinical trial design is essential for accurate assessment of the toxicities associated with these new and challenging treatment paradigms, and to ensure that the maximum impact on therapeutic index is achieved [25], [26].

The CONCORDE trial is, to our knowledge, the first phase I platform study for the safety assessment of multiple novel drug-RT combinations in LA-NSCLC and aims to inform how DNA damage response inhibitors (DDRi) can be combined with radical RT in patients unfit for concurrent CRT [27]. Five components of the DNA damage signalling pathway will be targeted using novel systemic agents, in combination with the international RT dose fractionation of 60 Gy delivered in 2 Gy once-daily fractions [6].

To exploit the rapid proliferation rates, aberrant DNA repair and reliance on the G2/M checkpoint commonly observed in NSCLC, we aim to evaluate DDRi directed at PARP, ATR, Wee1, ATM and DNA-PK in the first instance [28]. As non-malignant cells possess a functional G1/S checkpoint, in contrast with most NSCLC, it is proposed that selective tumour radiosensitisation can be achieved by abrogation of the G2/M checkpoint.

The potential targets are extensively reviewed as radio-sensitisers in Chalmers et al [26] but are briefly summarised here. PARP (poly-ADP ribose polymerase) enzymes are involved not only in repair of single strand DNA breaks but are key regulators of DNA damage repair [29]. Most clinical evidence for PARP inhibition is in the setting of BRCA-mutant malignancies [30], [31], [32], but early-phase trials in combination with radiotherapy for head/neck cancer were encouraging [33] and studies are planned in glioblastoma [20]. The ATR (ataxia telangiectasia and Rad3 related) and ATM (ataxia telangiectasia mutated) kinases are reportedly crucial for the repair of double-strand DNA breaks and are attracted to the break sites to enable homologous recombination [29]. Wee1 is a negative regulator at the G2-M checkpoint amongst other DNA damage response duties [29]. DNA-PK (DNA-dependent protein kinases) protect exposed DNA strands and so are particularly important in the non-homologous end joining DNA repair process [29].

Candidate compounds have been selected based on their dose enhancement factors in preclinical NSCLC models and emergent clinical data from other tumours, either as single agents or in combination with radiation and/or cytotoxic chemotherapy [33], [34], [35]. Given the significant diversity displayed in radiosensitising activity and effects on normal tissues [36], which are both dose and schedule dependent, the CONCORDE study has been designed to minimise risks and maximise clinical benefits. We will employ cautious drug dose escalation, careful adherence to organ at risk dose constraints and a Time To Event Continual Reassessment Method (TiTE-CRM) model to capture late toxicity whilst allowing efficient recruitment and dose escalation across five treatment arms, incorporating a concurrent control arm.

## 2 Methods

### 2.1 Study design

CONCORDE was developed by a national collaborative of clinicians, scientists, biostatisticians and industrial partners, under the auspices of the National Cancer Research Institute (NCRI) Clinical and Translational Radiotherapy Research Working Group (CTRad) and the NCRI Lung Group, with patient and public involvement embedded from study conception. The trial is sponsored by University of Leeds (MO20/118073) and funded by Cancer Research UK (A28890) and industry partners. The study complies with Research Governance Framework for Health and Community Care, the British Good Clinical Practice regulations and the Declaration of Helsinki, and is registered on the European Clinical Trials Database (EudraCT No 2020-000206-28).

**Fig. 1:**
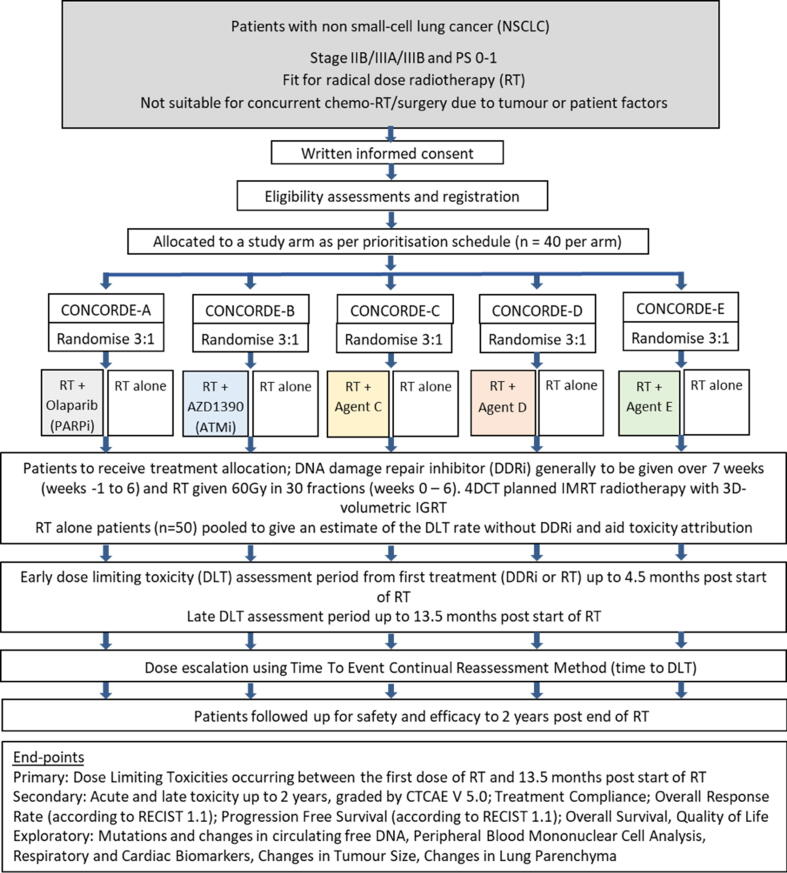
CONCORDE study summary.

CONCORDE is a randomised, open-label, phase Ib, multi-institution, multi-arm clinical trial seeking to determine the safety profile of multiple DDRi agents in combination with fixed-dose radical RT in LA-NSCLC (see Fig. 1). The platform nature engenders clinical trial efficiency with multiple parallel arms running simultaneously for different investigational medicinal products (IMPs). Accommodating the collection of side effects at later time points whilst avoiding stagnancy in accrual, use of the TiTE-CRM will further enhance study yield through continued enrolment while previous patients remain under follow-up [37]. CONCORDE integrates a calibration/control cohort of patients treated with RT alone to ensure that the safety data in the combination arms are interpretable. Eligibility criteria are listed in Table 1.

**Table 1:** Inclusion and exclusion criteria for CONCORDE.

*Inclusion Criteri*a	*Exclusion Criteria*
• Histologically or cytologically confirmed NSCLC • Unsuitable for concurrent CRT/surgery due to tumour or patient factors • Stage IIB and IIIA/IIIB (TNM 8 [38]) planned to receive radical RT +/- induction SACT • <8 weeks from previous SACT to the start of RT • Life expectancy estimated to be >6 months • Performance status (ECOG [39]) 0 or 1 • Medical Research Council (MRC) dyspnoea score [40] < 3 • Forced expiratory volume in 1 s ≥ 40% predicted • Diffusing capacity of the lungs for carbon monoxide ≥ 40% predicted • No prior thoracic RT (excluding breast RT, providing minimal overlap in RT volumes) • Adequate haematological, hepatic and renal function	• Mixed non-small cell and small cell tumours • Progressive disease during induction SACT • Participation in a study of an investigational agent/device < 4 weeks prior to treatment • Current/previous malignant disease which may impact on estimated life expectancy • History of interstitial pneumonitis • Prior treatment with pneumotoxic drugs within 1 year, or nitrosoureas with lung toxicity • Received a prior autologous or allogeneic organ or tissue transplantation • Cardiac history including uncontrolled ventricular arrhythmia, uncontrolled hypertension, uncontrolled atrial fibrillation, myocardial infarction within 3 months or long QT syndrome • Patients unable to swallow orally administered medications or chronic gastrointestinal disease likely to interfere with absorption of IMP in the opinion of the treating investigator • Prior RT where there is concern that the proposed treatment volume would overlap with a previously irradiated volume • Peripheral sensory neuropathy ≥ grade 2 • Active or prior documented autoimmune or inflammatory disorders • Exclusions as described in the relevant study arm protocol

### 2.2 Study objectives and end-points

Primary objective:

• Assess the safety and determine the recommended phase II dose (RP2D) of each DDRi used in combination with radical RT for patients with LA-NSCLC. The RP2D will be the dose level at which it is estimated that 25% subjects will experience dose limiting toxicities (DLT) (see Table 2) during the 13.5 month period from the start of RT

**Table 2:** Overview of dose-limiting toxicities.

Non-Haematological	Pneumonitis grade ≥ 4, or grade ≥ 3 for > 7 days Oesophagitis grade ≥ 4, or ≥ 3 for > 7 days Grade ≥ 3 nausea, vomiting or diarrhoea despite optimal medical management MRC dyspnoea score > 2 grades above baseline/CTCAE dyspnoea grade ≥ 3 for > 7 days Significant cardiac arrhythmia Any toxicity leading to interruption of RT for > 4 consecutive doses
Haematological	Neutropenia grade ≥ 4, or ≥ 3 with fever > 38.5 °C, or grade ≥ 3 for > 7 days Thrombocytopenia grade ≥ 4, or grade ≥ 3 for > 7 days or requiring transfusion Anaemia grade ≥ 3 or requiring a blood transfusion
Other	DLTs specific to particular DDRi agents as listed in the relevant study arm protocol Any other event, in the opinion of the SRC that is considered to be clinically significant and related to trial treatment

Secondary objectives include the description of:

• Safety profile (acute and late) using CTCAE v5.0 [41] and PROMs [42], [43]
• Treatment adherence for each DDRi in combination with RT
• Overall radiotherapy treatment time
• Best overall response using RECIST 1.1 criteria [44]
• Progression free survival (PFS) using RECIST 1.1 criteria [44]
• Overall survival

Exploratory objectives:

• Identify candidate biomarkers that could help select patients most likely to benefit from a combination of a specific DDRi and RT
• Investigate if imaging or circulating biomarkers of normal tissue damage could predict toxicity from DDRi-RT combinations early
• Investigate the impact of therapy with DDRi-RT combinations on the interplay between tumour and immune system

### 2.3 Radiotherapy

Patients will be planned using a 4D-computed tomography (CT) planning scan with intravenous contrast. Target volume delineation will be performed according the ICRU 62 guidance [45]. A motion-adapted gross tumour volume will encompass identifiable tumour and ‘CT/positron emission tomography (PET) positive’ lymphadenopathy. A 5 mm isotropic margin will be applied for the internal target volume and a further 5 mm margin for the planning target volume.

Treatment will be delivered with intensity modulated RT (>5-field) or volumetric modulated arc therapy and daily online cone-beam CT image guidance. RT will commence 7 days after the first dose of IMP and the total dose will be 60 Gy in 30 daily 2 Gy fractions over a period of 40 days based on EORTC and ICRU recommendations [46], [47]. The moderately hypofractionated regime commonly employed in the UK [48] was not chosen for the trial in order to minimise the possibility of acute normal tissue toxicities (i.e. oesophagitis and pneumonitis). RT quality assurance will be conducted through the UK National RT Trials Quality Assurance team. RT dosimetry and treatment data will be collected on all patients participating in the trial. Treatment start/stop rules are listed in Table 3.

**Table 3:** A summary of start/stop rules during treatment toxicity.

Scenario	Action
RT is suspended for RT-related toxicity	DDRi will be suspended until RT re-starts
RT is interrupted for logistical reasons (e.g. Linac breakdown)	DDRi will be continued during the interruption
RT is suspended for > 4 consecutive doses due to RT-related toxicity (DLT)	No further treatment with DDRi; RT will resume following toxicity resolution
DDRi is suspended due to DDRi-related toxicity with no increased RT-related toxicity	RT can continue; DDRi may be re-started at the next dose level down on resolution of toxicity to ≤ grade 1 or discontinued if assigned to dose level −1

### 2.4 Systemic therapy

Consented patients will be randomised between DDRi with RT or RT alone, on a 3:1 basis, meaning a maximum of 30 patients will be recruited to each experimental arm, with 10 controls each. Patients receiving RT alone will be pooled across arms as controls to provide contemporary toxicity data (≤50 patients for 5 treatment arms). DDRi therapy will be administered during the week before RT commences through to the end of RT or shortly after. DDRi schedules may vary between study arms e.g. intermittent dosing (such as alternate days). Induction SACT is permitted prior to trial enrolment but must be complete within 8 weeks of beginning RT. Consolidation immunotherapy will not be given at the start of the platform as the safety and efficacy in the setting of sequential chemoradiotherapy or radiotherapy alone is still being established (for example in PACIFIC-6 NCT03693300). However the platform is deliberately designed to adapt to changing standards of care, and may evaluate the safety of DDRi-RT and adjuvant immunotherapy combinations within its life-span.

### 2.5 Dose escalation

Toxicity will be assessed throughout treatment according to CTCAE V5 with weekly assessments until ≥ grade 2 toxicities have resolved to ≤ grade 1. DLTs will be monitored for up to 13.5 months post-start of RT in order to capture both the acute and long-term toxicities, subdivided into a ‘short DLT period’ and ‘long DLT period’:

• The short DLT period comprises DDRi and/or RT and up to 4.5 months from the start of RT. It is weighted for 90% in TiTE-CRM model, as 90% events are expected in this timeframe
• The long DLT period comprises 4.5 to 13.5 months from start of RT and will allow capture of later-onset DLTs. This long DLT period will be weighted as 10% in the model.

There will be pre-specified dose levels, including a −1 level for de-escalation if required. Each patient’s dose will be decided individually based on accumulated data available at the time of recruitment within the TiTE-CRM model. A separate, independent TiTE-CRM model will be used for each DDRi, with drug dose escalation decisions driven by the occurrence of DLTs and review by the safety review committee (SRC), chaired by an independent Thoracic Radiation Oncologist, and with independent clinicians, statisticians and patient representatives as key members. The pre-specified dose levels are based on existing early phase data in other disease sites. Escalations may consist of changes in either dose or schedule. Holds to recruitment and/or tightening of dose constraints to organs at risk may be advised by the SRC if required to evaluate potential excess toxicity.

Escalation of dose will be restricted until at least one patient has been followed up through the short DLT period. If the dose is reduced to dose level −1, recruitment will be restricted to approximately one patient per month, for at least the first 3 patients recruited, and additional pauses will be implemented prior to dose re-escalation. If the lower limit of the credible interval for the estimated probability of unacceptable toxicity is > 0.3 at dose level −1, or more than three DLTs are observed, that arm will be closed. If no DLTs are seen at any dose level, that trial arm will close to recruitment once 10 patients have completed the late DLT assessment period at the highest dose level, and that dose level will be deemed the RP2D.

### 2.6 Follow-up

Patients will be followed up until 2 years after the end of RT. Response to treatment and PFS will be assessed by CT at 1 month following completion of RT and subsequently at 3, 6, 12, 18 and 24 months, according to RECIST 1.1 where possible [44]. Robust patient reported outcome measures will be captured with validated questionnaires (EORTC-QLQ C30, EORTC-QLQ-LC29), and additional items from the EORTC-QLQ Item Library to cover potential novel agent toxicities, before randomisation, immediately post-treatment and during follow-up [42], [43].

### 2.7 Translational research

Patients will be consented for collection of residual archival tumour at baseline and disease progression for molecular analysis. Blood will be taken regularly for cfDNA analysis, T-cell repertoire analysis and circulating markers of cardiopulmonary toxicity. Imaging datasets including diagnostic imaging, planning scans, cone-beam imaging and response assessment scans will be collected for subsequent analysis.

## 3 Discussion

Despite RT offering the only curative option for a considerable proportion of patients with lung cancer, 5-year survival rates are dismal [5]. The investigation of drugs with synergistic potential in combination with RT has been neglected historically, due to the complexity of the necessary trials and a paucity of supporting preclinical data [49]. Conventional study designs are poorly suited to addressing such research questions [50]. Radiosensitisation with novel agents has significantly improved prognosis in a limited number of other tumours [51], [52], but such clinical benefit has not been demonstrated in the lung cancer population.

CONCORDE is the first platform trial of novel systemic treatment and RT in lung cancer patients [27]. As repair of radiation-induced DNA damage is thought to enable tumour cells to survive therapeutic radiation doses, there is a sound scientific rationale for the combination with DDRi, with supportive preclinical data [15], [16], [17], [18]. Related translational research will exploit the rich, prospective datasets generated, spanning tissue- and plasma-based genomics, immunobiology, biochemical pathology and radiomics.

This unique study is the result of close interdisciplinary working on a national level, most notably between oncology, biostatistics, science and industry. CONCORDE embodies the recommendations published in the NCRI CTRad consensus statement [25]. Furthermore, the novel multi-arm multi-stage trial infrastructure means that future patients wishing to participate in CONCORDE will benefit from up-to-date standard of care treatment as it evolves [53]. Standardised, state-of-the-art and contemporary RT within CONCORDE will provide the opportunity to comprehensively assess normal tissue toxicity in context, and randomisation will ensure that safety data are interpretable.

The CONCORDE platform provides a unique and hypothesis-driven opportunity to characterise the toxicity profile of novel radiosensitising drugs in NSCLC. It is anticipated that RP2Ds generated will enable the investigators to take candidate agents forward to dedicated randomised studies, and provide insights into the biology of radioresistance. The approach lends itself to future studies of novel drug-RT combinations, in NSCLC and other tumours.

## Declaration of Competing Interest

The authors declare that they have no known competing financial interests or personal relationships that could have appeared to influence the work reported in this paper.
